# Immunosenescence, aging and successful aging

**DOI:** 10.3389/fimmu.2022.942796

**Published:** 2022-08-02

**Authors:** Yunan Wang, Chen Dong, Yudian Han, Zhifeng Gu, Chi Sun

**Affiliations:** ^1^Department of Rheumatology, Affiliated Hospital of Nantong University, Medical School of Nantong University, Nantong, China; ^2^Department of Rheumatology, Affiliated Hospital of Nantong University, Nantong, China; ^3^Information Center, The First People’s Hospital of Nantong City, Nantong, China; ^4^Department of Geriatrics, Affiliated Hospital of Nantong University, Nantong, China

**Keywords:** immunosenescence, age-related diseases, successful aging, inflammation, centenarian

## Abstract

Aging induces a series of immune related changes, which is called immunosenescence, playing important roles in many age-related diseases, especially neurodegenerative diseases, tumors, cardiovascular diseases, autoimmune diseases and coronavirus disease 2019(COVID-19). However, the mechanism of immunosenescence, the association with aging and successful aging, and the effects on diseases are not revealed obviously. In order to provide theoretical basis for preventing or controlling diseases effectively and achieve successful aging, we conducted the review and found that changes of aging-related phenotypes, deterioration of immune organ function and alterations of immune cell subsets participated in the process of immunosenescence, which had great effects on the occurrence and development of age-related diseases.

## 1 Introduction

Aging, as a universal biological phenomenon, is an inevitable trend during lifespan and shows close effect on the immune system. The immune system is one of the most ubiquitous systems of the organism which can protect the human body from internal or external pathogens and interacts with neural, circulatory and other systems (1–3). Aging induces declining functions of the immune system, a process called immunosenescence, affecting the composition, quantity and function of immune organs, immune cells and cytokines (4). As a result of immunosenescence, the incidence of many age-related diseases is increased, including neurodegenerative diseases, cancers, cardiovascular diseases, autoimmune diseases and the COVID-19, ultimately resulting in organ failure and death (5–7). For a long time, immunosenescence has been considered harmful. However, later scientists revised the negative meaning because derogatory descriptions did not seize its essence. Immunosenescence is a multifactorial and dynamic complex phenomenon, which is shown as a lengthy adjusting and remodeled process existing in immune system during lifespan (8, 9). This review will compile the most recent researches of immunosenescence, including its relation with aging and its role in age-related diseases, thereby, providing scientists with theoretical rationales for intervention targets to aging.

## 2 The role of aging-related phenotypes in immunosenescence

**Figure 1:**
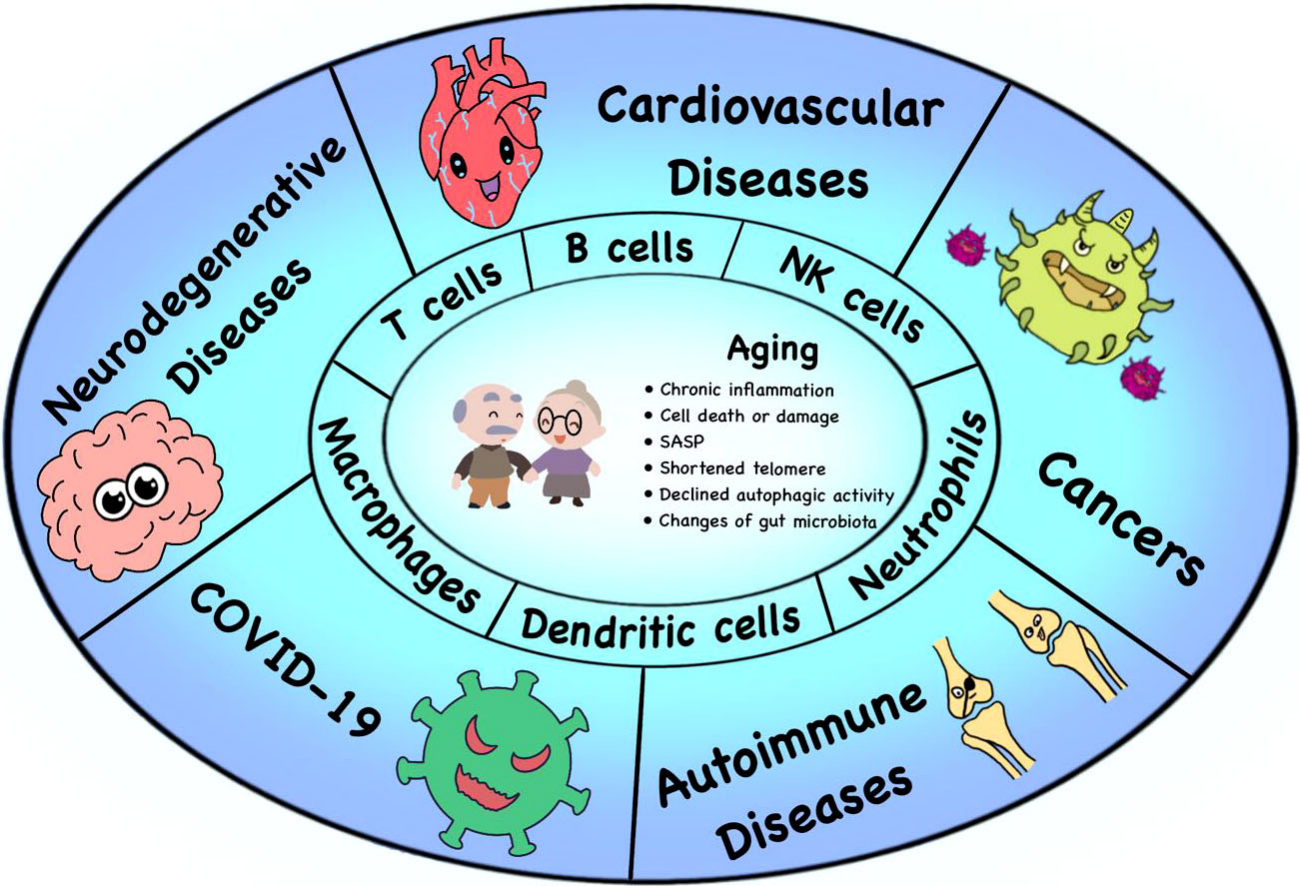
Immunosenescence and age-related diseases. Many factors lead to immunosenescence, including the accumulation of SASP, chronic inflammation, changes of gut microbiota, shortened telomere and declined autophagic activity. The process of immunosenescence causes a series of changes in immune cell subsets (especially T cells, B cells, NK cells, DCs, neutrophils and macrophages), thus leading to the occurrence of various age-related diseases, such as neurodegenerative diseases, tumors, cardiovascular diseases, autoimmune diseases and the COVID-19.

The molecular and cellular mechanisms of immunosenescence are mostly unclear. Many aging related phenotypes contribute to or are attributed to immunosenescence, including senescence-associated secretory phenotype (SASP), chronic inflammation, shortened telomere and decreased telomerase activity, and metabolic alternations, which are risk factors of age-related diseases **(** **Figure 1****)**.

### 2.1 SASP and immunosenescence

SASP is a pro-inflammatory phenotype including inflammatory factors, chemokines (CXCL, CCL), growth factors and extracellular matrix proteases, and accumulates persistently with the increase of senescent cells in various organs (10, 11). SASP is a universal characteristic of cell senescence. It spreads senescence in autocrine or paracrine manner and activates signal pathways (such as NF-kB, mTOR or p38MAPK) to affect cell microenvironment (11, 12). SASP induces inflammation, recruits immune cells and affects adjacent and distant cells or tissues (13). Actually, SASP has a close relationship with the immune system, for example, macrophage chemokines (MCP-1) are the main components of SASP (14). Certain SASP components are recognized by receptors on natural killer (NK) cells, T cells and monocytes/macrophages, and then affect other immune cells, which further release more proinflammatory cytokines and aggravate age-related pathology (13). SASP is a double-edged sword and different components induce different biological activities. It is beneficial that temporary secretion of SASP may be a danger warning to nearby cells and promote immune clearance of impaired cells (11). For example, SASP may attract innate and adaptive immune cells near tumor cells and precancerous lesions to resist cancer invasion (15). However, persistent secretion of SASP may cause chronic systemic inflammation and tissue damage and inhibit immune cell function in the elderly (11, 16). For instance, the SASP produced by precancerous hepatocytes may attract immature myeloid cells to inhibit NK cells and promote hepatocellular carcinoma progression, which seems to contradict the previous research (17). Why the SASP exhibits multiple and sometimes opposite effects, which remains to be explained.

### 2.2 Inflammation and immunosenescence

During aging, a state of chronic, low-grade, sterile inflammation has been known as inflammaging, which is essential to the aging process (18, 19). Many components (nucleic acids, mitochondrial DNA, cardiolipin, mitochondria and heat shock proteins) from cell death or damage increase with age, which may be recognized by innate immune receptors like toll-like receptors (TLRs), NOD-Like Receptors (NLR) and cGMP-AMP synthase (cGAS) and produce pro-inflammatory cytokines (20). SASP secreted from senescent adaptive immune cells, including T/B cells, may contribute to inflammation (21). Moreover, changes in the gut microbiota of the elderly may activate macrophages to a pro-inflammatory state and induce multiple inflammatory pathways, which is an important source of inflammation (22, 23). Macrophages initiate the inflammatory response and activate other immune cells by secreting inflammatory factors such as tumor necrosis factor (TNF)-α and interferon (IFN)-γ (7).

There is an imbalance between inflammatory and immune reactions in the process of aging, which reduces the efficiency of immune responses and creates an immunosuppressive microenvironment (24). Inflammatory mediators promote myelopoiesis and increase the immunosuppressive cells compensably, especially regulatory T (Treg) cells and M2 macrophages that secrete immunosuppressive factors, such as transforming growth factor-β (TGF-β), ROS and interleukin-10 (IL-10). IL-10 further support the proliferation and activation of Treg cells and M2 macrophages (25, 26). Interestingly, these factors also inhibit some immune cells and promote their immunosenescence, for example, TGF-β can inhibit the differentiation of helper T(Th) cells, reduce the cytotoxicity of CD8 T cells and NK cells and weaken the immune response of B cells (27). Once the balance is broken, a persistent increase of inflammatory response influences the activation of T/B lymphocytes, which is called immune paralysis and considered as one of the clinical features of immunosenescence (28). High levels of proinflammatory cytokines such as TNF-α damage human B cells and reduce the production of protective antibodies significantly (29, 30). Other cells such as macrophages and NK cells develop immune paralysis after long-term inflammation (31). Remarkably, inflammation is closely related to immunosenescence, but the debate continues about whether inflammation is a cause or result of immunosenescence.

### 2.3 Telomere and immunosenescence

The telomere biology system containing telomeres (DNA–protein complexes at the ends of chromosomes) and telomerases (reverse transcriptases that add DNA repeats to the ends of telomeres), is essential to maintaining the integrity of the genes and cells (32). In the immune system, the average telomere length and telomerase activity in lymphocytes decline with age (33, 34). The progressive telomere attrition from naive to effector memory cells shortens the telomere length, which may be resulted from mitochondrial stress. Moreover, shortened telomere could lead to DNA damage and cell cycle arrest, ultimately resulting in damaged cell function and inefficient pathogen clearance (35, 36).

Telomerase plays a vital role in immune activation, differentiation and immunosenescence through acting on key immunomodulatory factors such as NF-kB and β-catenin (37). The downregulation of telomerase activity is detrimental to the immune response and activates aging cells in the cloning process (37). The decrease of telomerase activity is usually accompanied by the increased intracellular ROS and the reduced CD28 expression. Senescent CD28-T cells with the shortest telomere length and the lowest telomerase activity produce decreased antiviral cytokines and increased pro-inflammatory cytokines (38, 39). CD28 costimulatory signal is necessary for upregulation of telomerase activity. A study measuring telomerase activity of T cells suggests that only telomerase activity of CD28+T cells is increased significantly under immune stimulation (40).

### 2.4 Metabolism and immunosenescence

It is clear that immune function is highly dependent on nutritional metabolism. The interaction between immune and metabolic process is termed as immunometabolism (41). The metabolic disorders of main nutrients (such as glucose, lipids and amino acids) in immune cells during aging lead to the dysregulation of nicotinamide adenine dinucleotide (NAD+) metabolism, activating inflammatory pathways and accelerating immunosenescence (42). With the increase of age, the level of glycolytic metabolism decreases and mitochondria energy metabolism is abnormal, which impairs T and B cell activation (43, 44). The NAD+ is a coenzyme which catalyzes cellular metabolic functions and converts to NADH. NAD+ decreases with age, which is resulted from reduced NAD+ biosynthesis, caused by chronic inflammation with increased oxidative stress and inflammatory cytokines, and increased NAD+ consumption, caused by DNA damage (45, 46). The reduction of NAD+ metabolism activates NLRP3 inflammatory bodies during age, which may be the key to inflammatory diseases (47). Proteostasis, an importance process to maintain protein structure and function, is compromised with age (48). Proteins are composed of a variety of amino acids that have a great impact on immune response, especially T cells (49). During aging, multiple proteins cannot be degraded and accumulate in tissues, contributing to the occurrence of age-related pathologies (50).

## 3 The contribution of immune organs on immunosenescence

### 3.1 Bone marrow involution with aging

Bone marrow contains haematopoietic stem cells (HSCs) and non-HSCs. HSCs are multifunctional immature cell populations that possess self-renewing capacity and give rise to all blood cells of immune system (51, 52). HSCs are decreased with aging and the senescent HSCs acquire increased DNA damage, dysfunctional function and myeloid bias, affecting the generation of naive T cells severely (53, 54).

HSCs show a more shift toward myeloid biased HSCs with age. The lymphoid-biased HSCs loss and the ability of common lymphoid progenitors (CLPs) to differentiate into the progenitor B cells is compromised so that the progenitor B cells decrease, which may be caused by changes in the different microRNAs (such as miR-29a, miR125b, and miR-150) and transcription factors (55–58). Other studies have shown that lymphoid-biased HSCs could be inhibited by TGF-β (59). Besides, the capacity of bone marrow stromal cells to release IL-7 (an important cytokine for survival and proliferation of B-lineage precursors) declines gradually, which is another mechanism for the development of progenitor B cells (60). However, senescence has no apparent effect on pro-B, pre-B and immature B cells (61).

Although age usually leads to decreased bone marrow cell density, the numbers of bone marrow resident NK cells (62) and macrophages (63) tend to increase in the elderly. NK cells are derived from bone marrow, which are characterized by high expression of specific markers CD16, CD56 or CD57. The HSCs are more likely to differentiate into NK cells, therefore, the frequency and absolute value of NK cells increase in the elderly (62). However, NK cells also display the loss of telomeres and the decrease of telomerase activity with age, which may lead to reduction of NK cell growth and proliferation (64). The aged macrophages have decreased ability to secrete inflammatory cytokines (63). Together, the composition of bone marrow and the ability to differentiate into functional immune cells are significantly impaired with age.

### 3.2 Thymic involution with aging

The thymus is a central lymphoid organ and responsible to produce naive T cells, playing an essential role in cellular and humoral immunity. Resulting from the loss of trophic cytokines such as IL-7 and decreased stem cell activity of medullary thymic epithelial cells, which are the main thymic stromal cells producing T cells, the thymus gradually degenerate that accompanies senescence (65, 66). T cells undergo T cell receptor (TCR) genes rearranging, positive and negative selection in the thymic cortex and medulla and become single positive naive T cells (CD4 or CD8) that are exported to the periphery (67–69). Thymic involution reduces naive T cells and TCR repertoire (70–72). CD8 T cells (especially cytotoxic CD8+ T cells) tend to loss much more severe than CD4 T cells which could be maintained by homeostasis and proliferation (73, 74). Thymic involution interferes with the negative selection resulting in the release of autoreactive T cells that become activated in the periphery and produce low-level proinflammatory cytokines (including TNF-α and IL-6) which lead to chronic low-grade inflammation and self-tissue damage (75). However, atrophic thymus balances the defective negative selection by enhancing thymic Treg (tTreg) cell production relatively in the elderly (76). The elderly suffer from high risk of cytomegalovirus (CMV) infection, which can accelerate immunosenescence by decreasing naive T cell diversity and exaggerating the cytokine storms (77). Interestingly, it is reported that well-preserved naive T cells can be found in centenarians (8).

### 3.3 Impaired peripheral lymphoid organs with aging

The peripheral lymphoid organs, predominantly referring to spleen and lymph nodes, provide the settled site for immune cells to be proliferation, maturation and differentiation, and participant in immune response. The peripheral lymphoid organs are also of vital importance in the interaction between T cells, B cells and antigen presenting cells (APC) (69). The aged spleen upregulates IL-6 expression, impairs the recruitment of T cells and inhibits phagocytosis of macrophages in the marginal zone (78–80). The key function of lymph nodes is to coordinate immune response. The lymphocytes in lymph nodes change significantly with age, including increased B cells and memory CD4 T cells, decreased γδ T cells, CD8 T cells, naive CD4 T cells, IgM-expressing B cells and follicular dendritic cells (FDCs) (81, 82). The lymph nodes show signs of aging, including permeability changes, senescent cell aggregation and inflammation, which may be disadvantageous for immune cell migration and recruitment (83), leading to decreased humoral immunity (84) and increased susceptibility to infections in the elderly (85).

Mucosa-associated lymphoid tissue (MALT) is also a part of peripheral immune organs, which is located on the surface of mucosal tissue and plays an important role in immune protection. Naive T/B cells and DCs in intestinal lymphoid tissue are reduced with age, which may explain the increased gastrointestinal cancers in the elderly (86). Therefore, age-related structural disorders of peripheral lymphoid organs and the changes of immune cells seem to be the main reasons for immunosenescence.

## 4 The alterations of immune cell subsets related to immunosenescence

**Figure 2:**
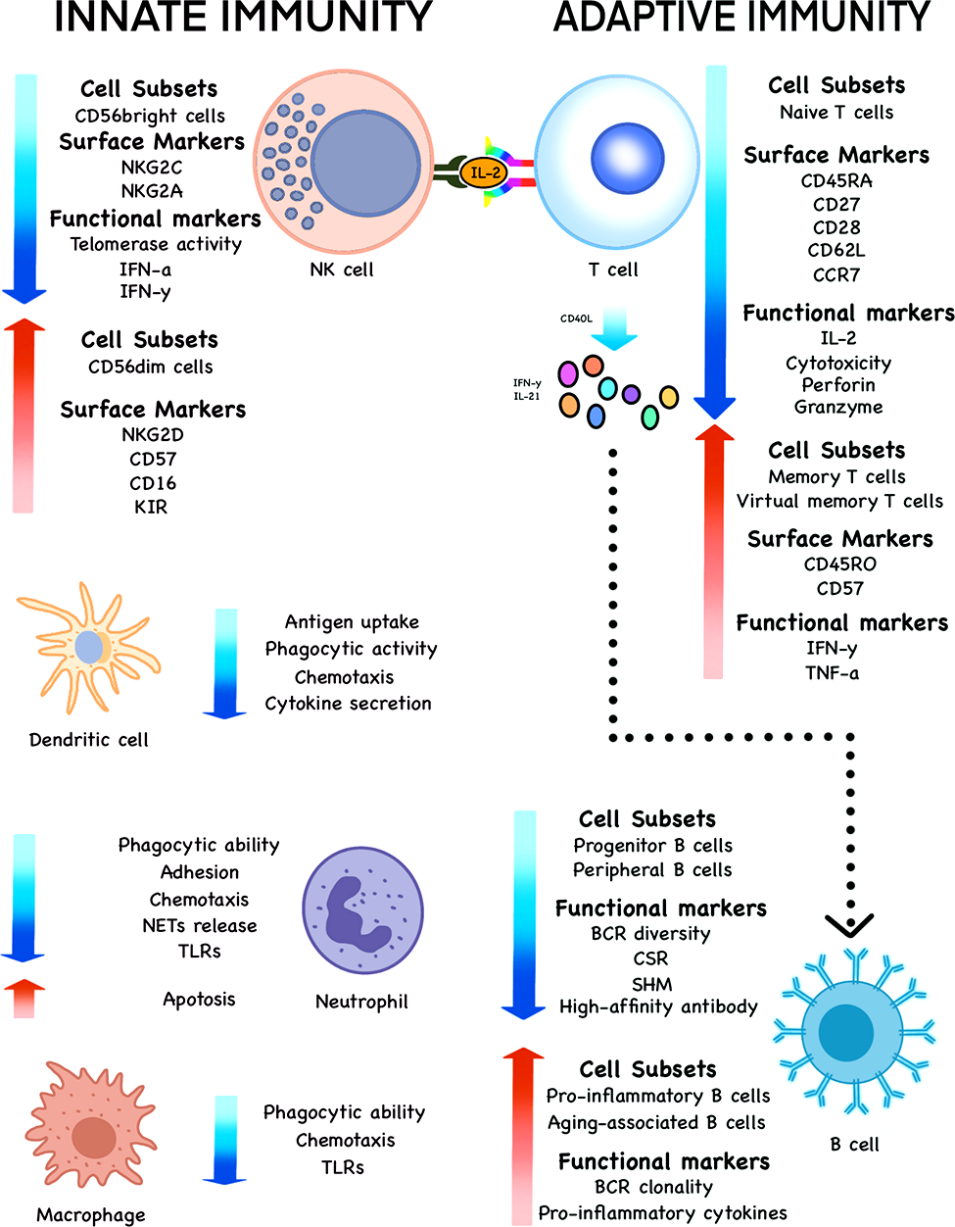
Changes of various cell subsets, surface markers and functional markers during immunosenescence. The subsets, phenotypes and functions of innate immune cells, such as NK cells, DCs, neutrophils and macrophages undergo significant changes with the increase of age in the elderly. The same is true of adaptive immune cells such as T and B cells.

Immunosenescence reflects the regulation of innate and acquired immune system, in which cell subsets, surface markers, quantity and function of immune cells, such as T cells, B cells, NK cells, DCs, neutrophils and macrophages, undergo a series of changes **(** **Figure 2****)**.

### 4.1 Adaptive immune cells

#### 4.1.1 T Cells

T cells, deriving from HSCs, mature in the thymus and migrate to peripheral lymphoid organs to expand and differentiate into memory and effector T cells under the antigenic stimulus, exerting a profound effect on immune system functions (87). T cells have specificity in recognition of foreign antigens and can be divided into several subsets including Th cells, cytotoxic T(Tc) cells and Treg cells, according to their different functions of immune response (88, 89). T cells undergo senescence with the loss of costimulatory molecules CD27 and CD28, the decreased growth factor IL-2 and the increased pro-inflammatory cytokine production (90–92). Senescent T cells can activate the inflammatory processes by contacting other immune cells, secreting pro-inflammatory cytokines or acting directly on the target tissues, eventually resulting in tissue damages and participation in the pathogenesis of aging (93). The genes related to leukocyte activation and immunity in aged memory T cells increase, which reduces the ability to recognize new pathogens and the response to vaccination, and increases risks for infection in the elderly (94).

##### 4.1.1.1 Helper T cells

Th cells, expressing the CD4 surface marker, coordinate the activities of the immune system by secreting cytokines or assisting other lymphocytes. Th cells are subdivided into Th1, Th2, Th9, Th17, Th22 and follicular helper T cells (Tfh). In aged humans, naive CD4 T cells tend to proliferate and differentiate into effector memory Th9 cells that secrete increased cytokines IL-9 due to the upregulation of the TGFβR3 receptor, leading to higher PU.1, BATF and IRF4 expression (95). The single-cell RNA sequencing uncovers that aging promotes T cells from naive to effector subtypes, among which Th1 and Th17 cell subsets are dominant. These subgroups are highly correlated with IL-6, IL-27 and IFN, which promotes chronic inflammation and declines immunity partly (96).

Tfh cells, presenting in lymphoid organs and peripheral blood, provide help for B cells that activate, differentiate and produce high-affinity antibodies by signals (such as ICOS, IL-12 and CD40L) (97). Reduced ICOS expression with aging could limit the number of Tfh cells (98). Increased pro-inflammatory cytokines IL‐12 with aging contribute to the formation of Tfh cells (99) and support differentiation of other Th cells, such as Th1 and Th17 cells (100). CD40L, highly expressed in Tfh cells, interacts with CD40 on B cells, which is vital for B cell immune response (101). Tfh cells express decreased CD40L in aged people, which reduces the assistance to B cells and contributes to decreased antibody titers after immunization (102).

##### 4.1.1.2 Cytotoxic T cells

Tc cells (also known as killer T cells), expressing the CD8 surface marker, are crucial in immune defense against harmful pathogens by secreting cytotoxic substances such as granzyme and perforin (103). As people get older, Tc cell proliferation is impaired along with the decreased naive cell marker (CD45RA and CD27), the lymphocyte adhesion molecule SELL (CD62L) and the lymphoid tissue homing chemokine receptor (CCR7), while the expression of memory cell marker CD45RO and the senescent marker CD57 increase (104). Cytotoxicity of Tc cells is reduced with aging, which decreases the killing effect on virus and increases disease risk in the elderly (61). Furthermore, cellular senescence is usually considered as the main mechanism of aging-related T-cell dysfunction (105). Tc cells also show cellular senescence characteristics, such as high levels of SA-βGal activity, p16INK4a, macroH2A and dysfunctional telomeres (106). Interestingly, aging might endow Tc cells with apoptosis resistance, for example, the antiapoptotic genes such as Serpina3g Id2 and S1pr5 are upregulated (94).

Some studies have confirmed a special kind of cells that express CD8 molecules and acquire memory phenotypes in the absence of antigen-specific immune responses, and are often termed virtual memory CD8 T (TVM) cells (107–110). These cells could patrol and monitor at the early stage, and disposal of pathogens during the effect-period, so they have been a bridge between innate and acquired immunity. TVM cells accumulate with age by cytokine stimulation (such as IL-4 and IL-15) but not by antigenic stimulation and exhibit characteristics consistent with senescence (111).

##### 4.1.1.3 Regulatory T cells

Treg cells, expressing inhibitory receptors such as programmed death 1 (PD-1) and cytotoxic T lymphocyte-associated antigen-4 (CTLA-4), make much difference to maintaining immune balance and limiting immunopathology by negatively regulating immune responses and secreting immunosuppressive cytokines TGF-β and IL-10 (112, 113). Treg cells are separated into natural Treg cells (nTregs) and induced Treg cells (iTregs or aTregs) (114). Despite thymic involution, the number and proportion of Treg cells increase in the elderly (115, 116), because Treg cells are derived from not only the thymus but also the differentiation of peripheral CD4+T cells and the proliferation of CD45RO+Treg cells (117), but their clonal diversity is reduced (118). A few studies have shown that Treg cell function decreases in the elderly, however, the overall data suggest that Treg function remains the same or even increases during aging, which is consistent with the fact that older individuals are more susceptible to infection and malignant tumors, while they are likely to develop autoimmune diseases due to Treg cell dysfunction (119).

The enhanced Treg cell function is related to increased expression level of forkhead box protein 3 (Foxp3) and hypomethylation of Foxp3 that is a master regulator of Treg cell function (120). Treg cells are more likely to be influenced by age-dependent autophagy inhibition due to more dependence on oxidative phosphorylation (121). Treg and Th17 cells are the key regulators of immune homeostasis. In the process of aging, the Th17/Treg imbalance that is driven driven by antigens or cytokines may result in abnormal immune response and the occurrence of various diseases (122, 123). The accumulation of CD8+Treg cells that mainly come from CD8+CD28-T cells, contributes to immune deficiency and declined adaptive responses with increasing age (124). Although a great progress has been made in the role of Treg cells related to immunosenescence, there are still many problems remaining to be resolved.

#### 4.1.2 B cells

B cells, a subset of adaptive immune cells, are crucially important in both cellular immunity and humoral immunity through secretion of antibodies, presentation of antigens and regulation of T cell functions (125). In addition to alterations in HSCs, intermediate and mature stages of B cell development also show a series of aging-associated changes. With increasing age, the proportion of peripheral B cells decreases (126). However, the number and frequency of pro-inflammatory B cells are expanded, which is largely because of increased pro-inflammatory signals CD40L, IFN-γ and IL-21 (127). Older adults display decreased repertoire diversity and increased BCR clonality (128). Aging also downregulates the expression of molecules with regard to immunoglobulin class-switch recombination (CSR) and somatic hypermutation (SHM) and reduces high-affinity antibody production (61). Neutralizing antibody responses and secretion of switched IgG play an important role during infection and vaccine efficacy. These shifts might increase the risk of bacteria and viruses in the elderly, such as the high hospitalization and mortality resulted from COVID-19 (129).

**Figure 3:**
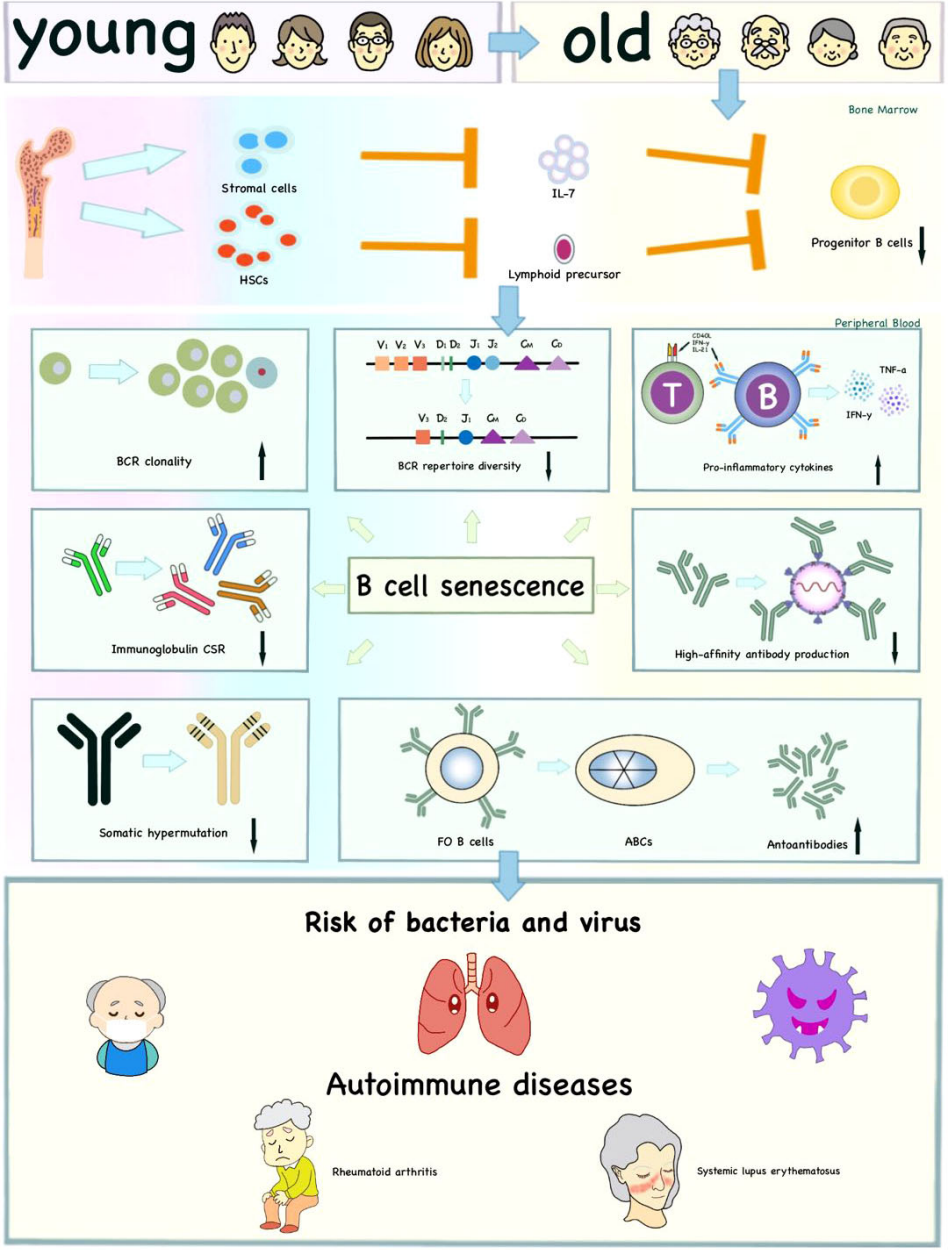
Effects of immunosenescence on B cells and age-related diseases. The reduced lymphoid output and impaired ability of bone marrow stromal cells to release IL-7 influence the production of progenitor B cells with age to some extent. Moreover, immunosenescence affects the intrinsic defects of B cells, including decreased BCR repertoire diversity, CSR, SHM, high-affinity antibody production and increased BCR clonality. The ABCs increase autoantibodies and pro-inflammatory cytokines. These age-related changes together increase the risk of infection and autoimmune diseases in the elderly.

It is reported that there is a novel B cell subset in human peripheral blood that accumulates with age, which is called the aging-associated B cells (ABCs) (130, 131). ABCs are generated by Follicular (FO) B cells *via* interactions between MHC class II and CD40/CD40L and distinguished from other B cells by their markers such as CD11b, CD11c and T-bet and signal transduction pathway such as TLR7 (132). ABCs displaying significant SHM and secreting autoantibodies, are closely related to autoimmune diseases (58, 131, 133). ABCs are reported to increase in elderly humans, especially in senile women with autoimmune diseases of lupus and rheumatoid arthritis (RA) (130) **(** **Figure 3****)**.

### 4.2 Innate immune cells

#### 4.2.1 NK cells

NK cells, as important components of human immunity, are a population of large granular lymphocytes with cytotoxic and immunomodulatory functions. Aging redistributes NK cells in numbers, phenotypes and functions (134).

NK cells contain two subpopulations, CD56^bright^ immunoregulatory cells and CD56^dim^ cytotoxic cells, which are differentially influenced by aging (135). While CD56^bright^ cells are decreased in old individuals, CD56^dim^ cells are expanded, suggesting the increase of NK cells with age results mainly from the expansion of CD56^dim^ cells. Therefore, aging induces NK cells from immature but robust cytokine producers CD56^bright^ NK cells to experienced and terminally differentiated CD56^dim^CD57^+^NK cells (136, 137). CD56^dim^CD57^+^cells are highly differentiated NK cell subpopulations, which have higher cytotoxic capacity, lower cytokine responsiveness and proliferation ability (138). During the aging process, CD56^dim^NK cells continue to differentiate and the expression of activated receptors natural-killer group 2 member D (NKG2D), immunoglobulin-like killer receptors (KIR), CD57 and CD16 increases, while the expression of activated receptors NKG2C and inhibitory receptors NKG2A decreases (139). Therefore, the cytotoxic function of NK cells is determined by the balance of activatory and inhibitory membrane receptor signals.

It has been shown that NK cell-activating cytokines (such as IL-2, IFN-α and IFN-γ) in old individuals are reduced, especially aged 75 to 85. IL-2 is an intermediary between innate and adaptive immunity and contributes to T cell and NK cell proliferation. Decreased cytokines (especially IL-2) may damage the immune response, leading to an increased incidence of infections among the elderly (140, 141). Moreover, aging may amplify sex difference in NK cells. Immature CD56^bright^NK cells and mature CD56^dim^NK cells in old women account for higher ratio. NK cells in female show stronger cytotoxicity, IFN-α responses to NKp46 crosslinking and MIP-1β production against external threats (142).

#### 4.2.2 Other cells

DCs, as central orchestrators of the immune response, are a bridge between innate and adaptive immunity. There is no significant effect on DC numbers and phenotypes in old humans, nevertheless, NK cells in skin and plasma cells (also called langerhans cells) are found to decrease. Besides, senescence could compromise the functions of DCs with regard to antigen uptake, phagocytic activity, chemotaxis and migration and cytokine secretion (143–145).

Neutrophils are a critical component of innate immunity (146). In aged individuals, a low-grade inflammatory state could lead to epigenetic changes in neutrophils which causes specific abnormalities in metabolism and function, such as diminished phagocytic ability (147), abnormal adhesion and chemotaxis (148), increased apoptosis (149), reduced NETs release (150) and TLR dysfunction (151).

Macrophages, as potent immunoregulatory innate immune cells, have a crucial effect on immune defense and regulation of inflammation (152). It has been highlighted that aging could disrupt circadian gene regulation and function of macrophages (153). Meanwhile, aging could be reversed by reprogramming glucose metabolism of macrophages and re-establishing youthful immune homeostasis (154).

## 5 The impact of immunosenescence on age-related diseases

### 5.1 Immunosenescence and neurodegenerative diseases

Alzheimer’s disease (AD), one of the most severe neurodegenerative diseases in the elderly, is characterized by elevated amyloid-β (Aβ) plaque deposition, neuroinflammation and brain-resident immune cells (microglia) (155–157). Elevated Aβ deposition can be captured by local APCs in the brain, which causes the activation and expansion of Aβ-reactive T cells, ultimately resulting in brain inflammation (158). AD patients have lower naive cells, higher memory cells and a significant telomere shortening of T cells (159, 160). The analysis of flow cytometry on peripheral blood of AD patients shows that CD8+T effector memory CD45RA+ (TEMRA) cells increase and TCR signaling is enhanced (161). Growing evidence indicates immunosurveillant CD8 T cells in the human brain, which represents central nervous system aging. Senescent T cells participate in enhancing proinflammatory effects of microglia, such as elevated proinflammatory cytokines, increased reactive oxygen species, dysfunctional lysosomal deposits, and eventually promote neuroinflammation (162, 163). Accumulated Treg cells in the peripheral immune system can impair the inflammation resolving immune cells’ infiltration into the central nervous system to suppress AD neuroinflammation, so targeting Treg cells has been an effective strategy to alleviate AD (157). These findings suggest immunity is involved in the development of neurodegenerative diseases, but further researches are necessary to study the interaction between senescent cell subsets and AD.

### 5.2 Immunosenescence and cancers

The declined immunity in older humans may increase the risk of cancers, which may be mediated by multiple cells (164, 165). In elderly people, immune function is obviously suppressed, which leads to increased tumor-infiltrating Treg cells, promoting tumor growth and metastasis (166, 167). For example, patients with breast cancer are found to exhibit immunosenescence, especially CD8+T cells (168). Macrophages contain two basic polarized states, proinflammatory classical activated (M1) and anti-inflammatory alternatively activated macrophages (M2). Tumor-associated macrophages (TAMs) usually display M2-like phenotypes to inhibit T cell activation and promote tumor metastasis, but macrophages can be polarized to kill tumor cells. Therefore, regulating the polarization of macrophages has been the potential effective strategies for anti-tumor therapy (169, 170). However, NK and B cells are less documented compared to T cells and macrophages of the tumor microenvironment, so extensive elucidations are expected in future.

### 5.3 Immunosenescence and cardiovascular diseases

Cardiovascular disease, which is associated with immunosenescence, has a high prevalence in the elderly population and is the leading cause of death among the elderly. T cells accelerate aging in patients with coronary heart disease and acute myocardial infarction, including telomere shortening and decreased expression of CD28 (171). Senescent T cells secrete pro-inflammatory cytokines, which activate macrophages and release metalloproteinases to degrade extracellular matrix (172). Senescent T cells also release cytotoxic components, such as perforin and granzymes, which damage endothelial cells and vascular smooth muscle cells directly (173). These results suggest senescent T cells may be involved in the pathophysiological process of cardiovascular diseases through inflammatory response and cytotoxicity.

Uncontrolled activation of the immune system has been resulted from the pathogenesis of hypertension, especially increased cytotoxic T cells (CD28- and CD57+) (174) and senescent NK cells that promote vascular remodeling and angiogenesis (175), which amplify the hypertensive action by releasing proinflammatory cytokines and cytotoxic mediators. With the increase of age, the accumulation of proinflammatory cytokines might increase monocyte specific TLR signaling, which is associated with the development of chronic heart failure (176, 177). Furthermore, senescent T cells are related to cardiovascular disease-related risk factors. For example, CD8+CD28-T cells accumulate in individuals with CMV infections that increase vascular inflammation and arterial blood pressure, promoting the occurrence of cardiovascular diseases (6, 178). In future, it needs to explore the impact of T cell senescence on cardiovascular diseases and determine whether senescent T cells are drivers or results of cardiovascular diseases.

### 5.4 Immunosenescence and autoimmune diseases

Immunosenescence is also associated with autoimmune diseases, especially RA. Immunosenescence is often accompanied by increased level of inflammation and both of them are the major contributors to age-related diseases. High levels of the pro-inflammatory cytokines, such as TNF and IL-6, lead to chronic inflammatory states for long time, which cause Th17/Treg imbalance and amplified immune response in the development of RA (179, 180). Nowadays, many studies have confirmed that RA patients exhibit premature immunosenescence, including thymus degeneration, clonal expansion of peripheral T cells and the loss of costimulatory receptor CD28 (181). Immunesenescence also deteriorates both articular and extra-articular manifestations, for example, CD4+CD28-T cells are especially marked in RA patients who have extra-articular inflammations or atherosclerotic diseases, and CD28-T cells are associated with poor cognitive functions of RA patients (181, 182). At present, the effects of T cell senescence on the occurrence and development of RA have been widely concerned, and other immune cells (such as Treg cells on RA, T/B cells on lupus or Sjogren Syndrome) have been reported, suggesting immunosenescence has an adverse effect on autoimmune diseases.

### 5.5 Immunosenescence and COVID-19

Due to aging-related immune changes, the pulmonary and systemic inflammatory responses are intensified, causing an increased risk of respiratory bacterial and viral infection such as influenza and the COVID-19 in the elderly (183, 184). The numbers of monocytes increase in the elderly, especially CD14 monocytes, which have high inflammatory gene expression and activate inflammatory signaling pathways, leading to the reduced ability of immune response (185). During the new crown epidemic, the relationship between immunosenescence and infections has received unprecedented attention. The COVID-19 has resulted in many deaths in the globe, which is characterized by hyperinflammation and cytokine storm severely involved in the lung, heart, kidneys and other multiple organs and systems (186). The elderly with COVID-19 show rapid clinical progress, high incidence and mortality (187–189), accompanying with heavy systemic inflammation and tissue damages, which would be related to immunosenescence, such as the decrease of plasmacytoid DCs (pDCs), alveolar macrophages and NK cells (183) and the increase of IGSF21+ DCs (187), neutrophils and CD14 monocytes (185). The immune cell sequencing shows that SARS-CoV-2 promotes immune cell polarization, mainly from naive T cells to memory/effector T cells, and gene expression associated with inflammation and cell aging (128). The COVID-19 can activate CD4+T lymphocytes to differentiate into pathogenic Th1 cells and produce cytokines (GM-CSF, etc), triggering cytokine storm (190). Severe SARS-CoV-2 patients show lessened number of CD4+ and CD8+T cells that express higher inhibitory receptors such as PD-1 and Tim-3, suggesting an exhausted status in T cells (185). Besides, the SARS-CoV-2 virus causes CD8+T cell senescence *via* TCR signaling and expressing CTLA-4 and TIGIT, and makes senescent CD8+T cells unable to release perforin and granzymes, which may explain susceptibility among the elderly (191). Actually, the decrease of adaptive immune response plays little role in COVID-19 mortality. Multiple organ failure and death are more associated with hyperfunctional natural immunity, high inflammation level and cytokine storm (186). To sum up, compromised immune function of the elderly is easier for the virus to spread and damage the tissues, which reinforces the necessity to resist immunosenescence and improve the immune function (such as the vaccine) to protect the body from the COVID-19.

Older people have a reduced response to vaccination because of immunosenescence, so it is important to strengthen the research on the safety and effectiveness of COVID-19 vaccine among the elderly population. However, the current studies on immune response in the elderly after COVID-19 vaccination have drawn different conclusions. The mRNA-1273 vaccine induces similar neutralizing antibody levels in different age groups (192). BNT162b1 and BNT162b2 vaccine induce similar neutralizing antibody titers between young and old people (193). ChAdOx1 nCoV-19 vaccine induces strong neutralizing antibody responses and cellular immune response against the spike glycoprotein at all ages, and the vaccine causes fewer side effects in the elderly (194). Despite the low neutralizing antibody level of the elderly, the mRNA vaccine produces similar immune response rates between old and young adults, which is opposite to other traditional vaccines. The mRNA vaccine, as a new type of vaccine, plays a much larger role in germinal center response, neutralizing antibody production, Tfh cell response and specific memory B cell response than traditional vaccine (195). Remarkably, mRNA vaccine enhances neutralizing antibody production after the second immunization, while traditional vaccine enhances non-specific antibodies (195). Furthermore, other studies have shown that the serum neutralizing antibody level of the elderly is still low after the first dose of BNT162b2 vaccine, but antibody immune response is improved significantly against variants of concern (VOC) after the second dose (196). The new crown inactivated vaccine booster makes SARS-CoV-2-specific memory B lymphocytes (about 7%) carry broad-spectrum neutralizing antibodies to provide effective protection against Omicron variant (197). Therefore, the elderly should not only be vaccinated actively, but also vaccinated with booster shots to resist diminished vaccine potency. Nowadays, mRNA vaccine has gradually replaced the traditional vaccine as the most popular vaccine for vaccinators of different ages (especially the elderly). However, vaccine induced protective mechanism of old vaccinators are still unclear, and more researches remain to be done.

## 6 Immunosenescence and successful aging

For a long time, immunosenescence has been considered harmful. However, it is noteworthy that immunosenescence is a remodeling and retuning process with increase in some new functions rather than complete decline of immune function (9). Serum levels of lgG and lgA are increased with age, which is conducive to protecting against viral and bacterial infections effectively in older people (198). Although the generation of naive T/B cells continues to decline, the adaptive immune system adjusts to age-related changes and protects the body from most pathogens. Only later in life does the immune function decline gradually, which increases morbidity and mortality in the elderly (199). But not all older people suffer from age-related diseases, centenarians can delay the aging process and live up to the limits of human life. Centenarians have a large quantity of anti-inflammatory molecules, such as TGF-β1, IL-10 and IL-1 receptor antagonist (IL-1RA), to counterbalance increased inflammatory molecules, such as IL-1β, IL-6, TNF-α, IL-8, C-reactive protein (CRP) and CXCL9, achieving a dynamic balance between pro-inflammatory and anti-inflammatory levels (8). In addition, telomere length and telomerase activity are higher in centenarians (200).

**Figure 4:**
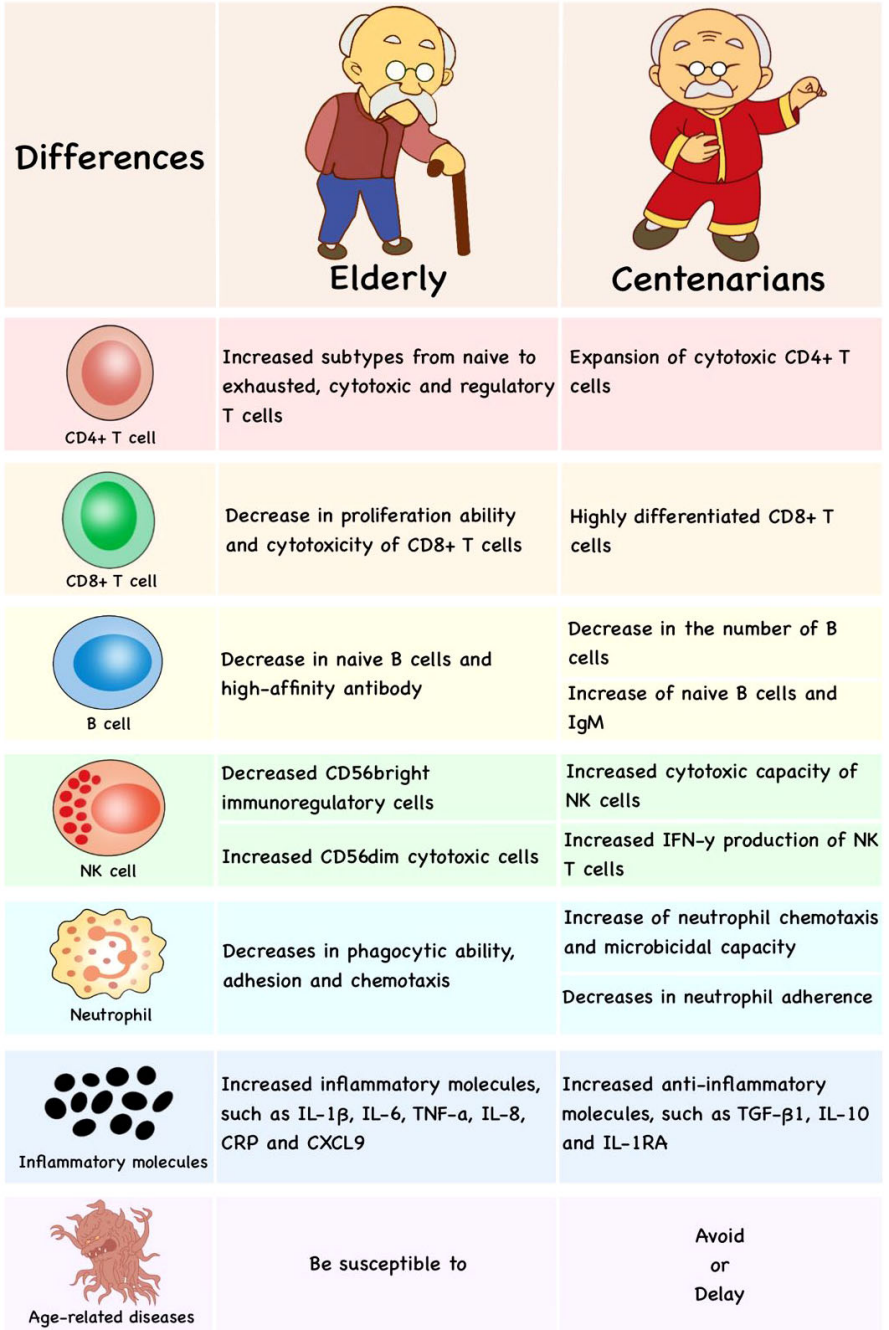
The differences in immune cells of published researches between the elderly and centenarians. Compared with the elderly, centenarians have more anti-inflammatory molecules, cytotoxic CD4+T cells, highly differentiated CD8+T cells, naive B cells and well-preserved NK cells, which would be the hallmark of successful aging.

In centenarians, the degradation of immune function is not obvious. Interestingly, the expansion of cytotoxic CD4+ T cells has been found in supercentenarians and makes them resistant to diseases (201). CD8+T cells of centenarians are highly differentiated with decreased CD28 expression (which are also called CD8+CD28−, CD8+KIR+, NK-like CD8+ or innate CD8+T cells) (202) and higher CD45RA expression (203). In centenarian offspring, the number of B cells decreases significantly, but naive B cells and IgM increase, which might be one of the reasons for resisting infection and prolonging the lives (204). The cytotoxic capability of NK cells in centenarians (up to about 55%), which is very similar to the young groups (about 63%), is higher than that in middle-aged groups (about 33%) (205). NK T cells bearing γδ TCR show higher cytotoxicity and IFN-γ production in centenarians, which is beneficial to fighting diseases and successful aging (141). Moreover, neutrophil chemotaxis and microbicidal capacity and lymphoproliferation are higher in centenarians, while neutrophil and lymphocyte adherence are lower (206). Therefore, anti-inflammatory molecules, cytotoxic CD4+ T cells, naive B cells and well-preserved NK cells would be the hallmark of successful aging **(** **Figure 4****)**.

## 7 Therapeutic strategies for successful aging

There are currently several strategies to deal with senescence and senescent cells. First of all, rejuvenation of old HSCs may be an effective therapeutic strategy to restore the balance between myeloid and lymphatic systems and the numbers of T and B cells (207). The involution of the thymus is one of the main features of aging, which might lead to the decrease of T cells, so restoring the structure and function of the aging thymus could reverse immunosenescence (208). Thymo-stimulatory property of IL-10, leptin, keratinocyte growth factor (KGF) and thymic stromal lymphopoietin (TSLP) may contribute to immune reconstitution of the elderly (69). IL-7 is a crucial cytokine for T cell development, so IL-7 treatment promotes the expansion of peripheral T cells and the diversity of TCR (209, 210). Telomerase is a significant component for T cell development, so upregulation of telomerase expression enhances T cell immune response and prolongs lifespan (211, 212).

Senescent cells cause immune cells dysfunction by recruiting SASP, which is connected with many chronic diseases, so clearing senescent cells is of great importance. Senotherapeutic strategies contain two types: senolytics (removing senescent cells selectively) and senomophics (changing senescence phenotypes) (213). A novel senolytic agent ABT-263 causes apoptosis in senescent cells by targeting Bcl-2 family members (a negative regulator of apoptosis) (214). A FOXO4 peptide induces apoptosis of senescent cells by interfering the FOXO4-p53 interaction (215). A component of grape seed extract procyanidin C1 (PCC1) is a natural senolytic agent and extends lifespan in mice (216). Nowadays immunotherapy is a promising therapeutic strategy against senescent cells (217). Modified T cells that express a chimeric antigen receptor (CAR) have been applied in cancer treatments successfully (218), based on which, engineering CAR T-cells with NKG2D receptors contributes to recognizing and eliminating senescent cells with NKG2D ligands (219). Chemotherapeutic agents (doxorubicin, melphalan and bortezomib) enhance the killing effect of NK cells to clear senescent cells by upregulating the expression of NKG2D receptors on tumor cells (220). The main culprit of senescent cells is the SASP, therefore, the way to prevent cell senescence is to control or neutralize SASP by blocking main upstream regulators (such as GATA4, NF-κB and BRD4) or using targeting drugs (221). Rapamycin, a common inhibitor of mTOR, prevents senescence through decreasing markers of senescence in peripheral T cells and inhibiting SASP regulators (222–224). Metformin has been known to reduce SASP by modulating NF-κB signaling and delay the aging process (213).

Immune checkpoint blockade (ICB) therapy has been applied in cancers, for example, PD-L1 and IDO may restrain T cells immunity (225, 226). However, data on the safety and toxicity of ICB therapy are limited, so further researches are required to evaluate the therapeutic effects of ICB especially on the elderly. Treg targeted therapy is vital for cancer therapy and the treatment of autoimmunity, but sometimes has some risks. For example, Treg targeted therapy treats autoimmune diseases through inhibiting autoreactive immune components, but increases tumor immune escape and the risk of cancer, which results in the complexity of Treg targeted therapy (227). Growth differentiation factor 15 (GDF15) is a stress response gene caused by mitochondrial dysfunction and maintains the immunosuppressive function of Treg cells, so the intervention of GDF15 may improve the immune function of the elderly (228). Notably, rituximab is an anti-CD20 monoclonal antibody, which may inhibit pro-inflammatory B cell subsets such as ABCs, combating age-related autoimmune diseases. Fruit and vegetables, richen in carotenoids, increase the number of NK cells and the function of Th cells, ultimately enhancing the immune function (229). Vitamin E supplementation strengthens the function of T cells by reducing PGE2 production in macrophages, having a beneficial effect on healthy elderly (230, 231). It has also been shown that exercise decreases the number of Th17 cells and inflammatory markers and increases the level of IL-7, thymic function and autophagy activity (36, 232), suggesting the contribution of diet and exercise for the plasticity of aging.

The more we understand the cellular and molecular mechanisms of aging, the more opportunities we create to intervene aging and age-related diseases. In conclusion, the evidence suggests that targeting drugs and a good lifestyle together help to boost the immune system and enable the elderly to live longer and heathier.

## 8 Conclusion

Immunosenescence is a complex and varied process of immune system, which participates in many age-related diseases, with the alteration of immune cell subsets, cytokine secretion and the defect in cell function and quantity (176, 233, 234). It is of significant importance to explore the molecular and cellular mechanisms of immunosenescence with single-cell techniques to dissect some phenomena deeply and systematically (235). Moreover, it is necessary to distinguish markers of immunosenescence, quantify the immunity and establish normal reference range of immune cells among individuals at different age, which contributes to screening, preventing and intervening diseases even at subclinical stage. Therefore, it is obviously necessary to find novel targets and therapy for immunosenescence, for example, vaccines and microbiome regulation, to decline the negative effects of immunosenescence and promote successful aging.

## Author contributions

ZG and CS contributed to conception and design of the study. YW and CD complete the review of literature and wrote the first draft of the manuscript. YH contribute to the graphic visualization. All authors contributed to manuscript revision, read, and approved the submitted version.

## Funding

This work was supported by National Natural Science Foundation of China (grant number 82071838 and 82101891), Jiangsu province senile health scientific research project (grant number LR2021035) and Science and technology Project of Nantong City (grant number JC2021119) and Postgraduate Research & Practice Innovation Program of Jiangsu Province (grant number SJCX21_1459 and KYCX21_3106).

## Conflict of interest

The authors declare that the research was conducted in the absence of any commercial or financial relationships that could be construed as a potential conflict of interest.

## Publisher’s note

All claims expressed in this article are solely those of the authors and do not necessarily represent those of their affiliated organizations, or those of the publisher, the editors and the reviewers. Any product that may be evaluated in this article, or claim that may be made by its manufacturer, is not guaranteed or endorsed by the publisher.
